# Differentiating among incretin-based therapies in the management of patients with type 2 diabetes mellitus

**DOI:** 10.1186/1758-5996-4-8

**Published:** 2012-03-05

**Authors:** Michael Cobble

**Affiliations:** 1 Canyons Medical Center, 9355 S 1300 E, Sandy, UT 84094, USA

**Keywords:** type 2 diabetes, exenatide, liraglutide, sitagliptin, saxagliptin, linagliptin, efficacy, safety

## Abstract

The glucagon-like peptide-1 receptor (GLP-1R) agonists and dipeptidyl peptidase-4 (DPP-4) inhibitors have become important options for the management of patients with type 2 diabetes mellitus. While the GLP-1R agonists and DPP-4 inhibitors act on the incretin system to regulate glucose homeostasis, there are important clinical differences among the five agents currently available in the U.S. For example, the GLP-1R agonists require subcutaneous administration, produce pharmacological levels of GLP-1 activity, promote weight loss, have a more robust glucose-lowering effect, and have a higher incidence of adverse gastrointestinal effects. In contrast, the DPP-4 inhibitors are taken orally, increase the half-life of endogenous GLP-1, are weight neutral, and are more commonly associated with nasopharyngitis. Differences in efficacy, safety, tolerability, and cost among the incretin-based therapies are important to consider in the primary care management of patients with type 2 diabetes mellitus.

## Introduction

Treating patients with type 2 diabetes mellitus (T2DM) can be very challenging. Fortunately, new treatment options for T2DM, such as incretin-based agents, provide new opportunities to bring the disease under control, and perhaps slow its progression. More recently, focus has been placed on 'treating to target' glucose approaches rather than waiting for progressive glucose failure. The goal of the treat-to-target approach is to achieve safe glucose targets for each individual with a combination of early lifestyle and pharmacologic therapies. As such, it is important to work with each patient to develop and initiate a lifestyle and pharmacologic treatment plan at the time of diagnosis of T2DM to achieve the glycemic target--generally an A1C < 7.0% [1], within 3 to 6 months. The second and very important part of the treat-to-target approach is to modify treatment as needed to maintain the A1C at the target level [2]. Modifying treatment is, however, often challenging because of hypoglycemia, weight gain, intolerable adverse events, even access to and affordability of newer agents, as well as clinical inertia. These and other glycemic and non-glycemic factors were considered by the American Diabetes Association/European Society for the Study of Diabetes (ADA/EASD) [2] and by the American Association of Clinical Endocrinologists/American College of Endocrinology (AACE/ACE) [3] when developing their 2009 guideline recommendations. Both groups concluded that, based upon their unique physiologic activity, efficacy, nonglycemic benefits, and safety profiles, agents which act on the incretin system--the glucagon-like peptide-1 (GLP-1R) agonists and dipeptidyl peptidase-4 (DPP-4) inhibitors--are important options for the management of patients with T2DM. An agent in each class has now been FDA-approved since 2005 and 2006. (Table 1)

**Table 1:** Comparison of GLP-1R agonists and DPP-4 inhibitors.

	GLP-1R Agonists	DPP-4 Inhibitors
Agents currently available in U.S. with dosing information (normal renal function)[31-35]	• Exenatide 5-10 mcg SC BID • Liraglutide 1.2-1.8 mg QD	• Sitagliptin 100 mg PO QD • Saxagliptin 2.5-5 mg PO QD • Linagliptin 5 mg PO QD

**Benefits**		

Reduction in A1C level*[22-24,26,29,36-45]	0.5%-1.5%	0.5%-0.9%

Reduction in fasting plasma glucose*[29,39-41,49-51]	↓7 to 74 mg/dL	↓11 to 29 mg/dL

Reduction in postprandial glucose*[9,27,51,54,55]	↓41 to 47 mg/dL	↓49 to 68 mg/dL

Weight effect [14,22,24,26,29,37,39-41,44,45,49,50,52,60]	↓1-4 kg	↓0.9 to ↑1.4 kg

Effect on triglycerides [24,29,36,37,39,41,49,60,62]	↓12-40 mg/dL	↑16 mg/dL to ↓35 mg/dL

Reduction in systolic blood pressure [13,14,24,29,36,37,39,41,49,60,62]	↓1-7 mm Hg	0 to ↓3.9 mm Hg

May improve markers of pancreatic β-cell function (such as homeostasis model assessment-β-cell function, fasting insulin, fasting proinsulin to insulin ratio, fasting C-peptide)[8,13,22-24,26,30]	✓	✓

**Disadvantages**		

Incidence of mild/moderate hypoglycemia**[9,10,24,26,36-39,41,43-45,52,55,64]	0%-12%	0%-4%

Nausea [13,33-35]	26%-28%	0-1%

Hypersensitivity reactions [33-35]	Rare (exenatide)	✓

Antibody formation [31-35,79,80]	30-67% E; 8% L	NR

*As monotherapy or as add-on therapy.

**Generally included asymptomatic hypoglycemia or symptomatic hypoglycemia with blood glucose < 55 mg/dL not requiring third-party assistance.

BID, twice daily; NR, not reported; PO, orally; QD, once daily; SC, subcutaneously

**Figure 1:**
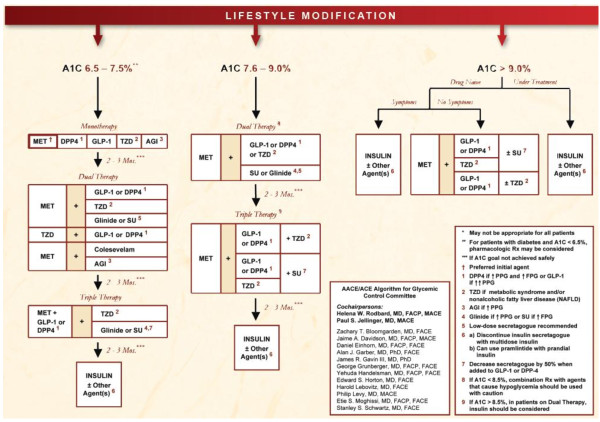
**AACE/ACE diabetes algorithm for diabetes control**. Algorithm for the metabolic management of type 2 diabetes. Lifestyle modification is a component of treatment for all patients. Interventions are stratified based upon the current A1C level and whether the patient is receiving treatment or is drug naïve. Medication choices are prioritized according to safety, risk of hypoglycemia, efficacy, simplicity, anticipated degree of patient adherence, and cost of medications. Only combinations of medications approved by the US Food and Drug Administration that provide complementary mechanisms of action are listed. It is essential to monitor therapy with A1C and self-monitoring of blood glucose and to adjust or advance therapy frequently (every 2 to 3 months) if the appropriate goal for each patient has not been achieved. [Reprinted from Endocrine Practice, Volume 15, Rodbard HW, Jellinger PS, Davidson JA, Einhorn D, Garber AJ, Grunberger G *et al. *Statement by an American Association of Clinical Endocrinologists/American College of Endocrinology consensus panel on type 2 diabetes mellitus: an algorithm for glycemic control, Pages 540-559, Copyright 2009, with permission from the American Association of Clinical Endocrinologists.]

**Figure 2:**
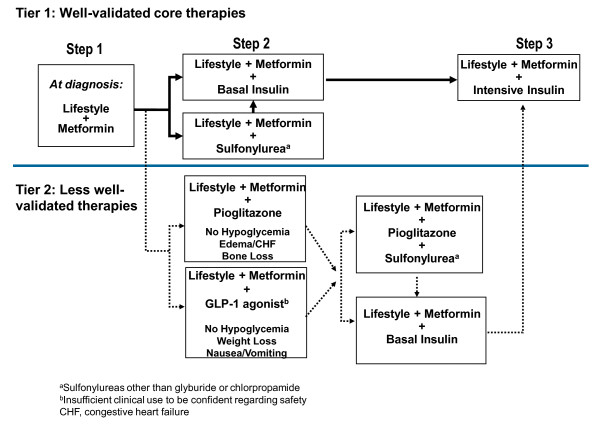
**ADA/EASD algorithm for the management of patients with type 2 diabetes**. Algorithm for the metabolic management of type 2 diabetes. Reinforce lifestyle interventions at every visit and check A1C every 3 months until A1C is < 7% and then at least every 6 months. The interventions should be changed if A1C is ≥ 7%. [Diabetes Care by American Diabetes Association. Copyright 2009 Reproduced with permission of American Diabetes Association in the format journal via Copyright Clearance Center]

The AACE/ACE guidelines, for example, state that the GLP-1R agonists and DPP-4 inhibitors are options as monotherapy for patients with an A1C of 6.5% to 7.5%, as well as in combination with other glucose-lowering agents for patients with an A1C > 7.5% (Figure 1). In this latter situation, the GLP-1R agonists are given a higher priority than the DPP-4 inhibitors because of the greater effect of the GLP-1R agonists in reducing postprandial glucose excursions and their potential for inducing substantial weight loss. The ADA/EASD recommendations take a different approach recommending the GLP-1R agonists (and thiazolidinediones) as less-validated alternatives to insulin or sulfonylurea as add-on therapy to lifestyle management and metformin (Figure 2). The DPP-4 inhibitors are appropriate for selected but unspecified patients according to the ADA/EASD recommendations, which were published in early 2009.

### Physiologic Actions

The multifactorial nature of the pathophysiology ofT2DM presents several options for treatment, some of which are not addressed by standard glucose-lowering therapies. Standard glucose-lowering therapies generally improve insulin sensitivity, stimulate insulin secretion, and/or decrease hepatic glucose production. The incretin system exerts somewhat unique actions upon glucose homeostasis. In fact, the incretin system may be responsible for up to 70% of insulin secretion in response to oral glucose or a meal in healthy individuals [4].

Of the two principal incretin hormones, GLP-1 plays a more important role in T2DM since the insulinotropic action of glucose-dependent insulinotropic polypeptide (GIP) is essentially lost in persons with T2DM, while the insulinotropic activity of GLP-1 following administration of pharmacological doses of GLP-1 is preserved [5].

Extensive preclinical investigations with GLP-1 and subsequent clinical investigations with the GLP-1R agonists and DPP-4 inhibitors in humans, including patients with T2DM, have been undertaken to determine the actions of each of these agents in regulating glucose. As expected from studies investigating the actions resulting from administration of GLP-1, stimulation of the GLP-1 receptor directly with a GLP-1R agonist or indirectly with a DPP-4 inhibitor (by inhibiting enzymatic degradation of endogenous GLP-1) serves to increase insulin secretion in a glucose-dependent manner through direct activation of pancreatic islet β-cells [6-10] and to inhibit glucagon secretion in a glucose-dependent manner (ie., only during hyperglycemia) through direct activation of pancreatic islet α-cells [6,8,9,11-14]. Furthermore, administration of GLP-1 or a GLP-1R agonist (but not a DPP-4 inhibitor) has been shown to slow gastric emptying [8,15] and to promote satiety [8,16,17], which leads to weight loss, although neither of these effects is required for the glucose-lowering benefits of the GLP-1R agonists. This difference is thought to be due to the 60 pmol/L pharmacologic level of GLP-1 activity achieved with administration of a GLP-1R agonist [18], which is associated with nausea, compared to the 10 pmol/L physiologic level of endogenous GLP-1 activity achieved from administration of a DPP-4 inhibitor [19].

In addition, exposure to GLP-1 in cell culture [20] or administration of GLP-1 in rodents [21] has been found to promote proliferation, increase differentiation, and prolong survival of pancreatic β-cells. These observations have prompted an assessment of the possible beneficial effects of the GLP-1R agonists and DPP-4 inhibitors on β-cell function in humans. While some trials have demonstrated improvement of surrogate markers of β-cell function, others have not [8,10,13,22-30]. Further investigation is ongoing.

GLP-1 is secreted by the L-cells of the small intestine in response to food ingestion, but undergoes rapid enzymatic degradation by DPP-4. As a consequence, injectable GLP-1R agonists were developed that resist degradation by DPP-4. Exenatide (for twice-daily administration) and liraglutide are the GLP-1R agonists that are currently available in the U.S. In addition, oral inhibitors of DPP-4 have been developed that prolong the action of endogenous GLP-1. Sitagliptin, saxagliptin, and linagliptin are the three DPP-4 inhibitors currently available in the U.S. The recommended maintenance doses in the current prescribing information are: exenatide 10 μg twice daily [31]; liraglutide 1.2 or 1.8 mg once daily [32]; sitagliptin 100 mg once daily [33]; saxagliptin 2.5 or 5 mg once daily [34]; and linagliptin 5 mg once daily [35].

### Findings from Clinical Trials

Incretin-based therapies have been investigated in numerous clinical trials involving patients with T2DM. These trials have typically involved patients with somewhat different characteristics at baseline. Results of trials that have compared an incretin-based agent with one or more non-incretin agents are described below to provide a general understanding of the glycemic and non-glycemic effects of the incretin-based agents. To gain a greater understanding of differences among incretin-based therapies, results of head-to-head trials comparing two incretin-based agents are also provided.

### Glycemic Effects

#### A1C

As a consequence of their physiologic actions to regulate glucose homeostasis, the GLP-1R agonists and DPP-4 inhibitors are effective in lowering blood glucose levels, with a more robust effect with the GLP-1R agonists (Table 1). When administered as monotherapy or in combination with metformin or other glucose-lowering therapy, the GLP-1R agonists reduce the A1C level by 0.5% to 1.5% [29,36-41] and the DPP-4 inhibitors by 0.5% to 0.9% [22-24,26,42-45]. Also, patients previously treated with diet and exercise alone have been observed to achieve a greater A1C reduction with liraglutide than those previously treated with glucose-lowering monotherapy (-1.6% vs -0.7%, respectively) [37]. Patients with a baseline A1C level ≥9.0% appear to experience a greater reduction in A1C with sitagliptin than patients with a baseline A1C level < 8.0% (-1.5% vs -0.6%, respectively) [22]. Similar results have been observed with exenatide [40], liraglutide (baseline A1C ≥10%) [13] and linagliptin [45].

Prospective head-to-head comparative trials have demonstrated differences among the incretin therapies with respect to their efficacy in reducing the A1C level as add-on therapy. In these trials, patients were those who had inadequate glycemic control with metformin-based therapy. In one trial after 26 weeks, exenatide 10 μg twice daily reduced the A1C by 0.8% compared to 1.1% with liraglutide 1.8 mg once daily (*P *< 0.0001) [13]. Another 26-week trial showed that liraglutide 1.2 or 1.8 mg once daily reduced the A1C by 1.2% and 1.5%, respectively, compared to 0.9% for sitagliptin 100 mg once daily (*P *< 0.0001 vs both liraglutide doses) [30]. After 52 weeks, the A1C reductions from baseline were 1.3% and 1.5% for liraglutide 1.2 and 1.8 mg, respectively, and 0.9% for sitagliptin (*P *< 0.0001 vs both liraglutide doses) [46]. A third trial involved initiation and stabilization of insulin glargine as add-on therapy. After 4 weeks, exenatide (5 μg twice daily for 2 weeks, then 10 μg twice daily for 2 weeks) lowered the A1C 1.8% compared to 1.5% for sitagliptin 100 mg once daily [47]. A fourth trial showed that after 18 weeks, sitagliptin 100 mg once daily lowered the A1C 0.6% compared to 0.5% for saxagliptin 5 mg once daily [48].

#### Fasting plasma glucose

Fasting plasma glucose (FPG) levels are also reduced when GLP-1R agonists or DPP-4 inhibitors are administered as add-on therapy to other glucose-lowering agents (Table 1). Specifically, reductions in FPG are observed with the addition of a GLP-1R agonist ranging from 7 to 74 mg/dL [29,39-41,49]. The head-to-head comparison of exenatide with liraglutide showed that liraglutide caused a significantly greater reduction of the FPG than exenatide (29 mg/dL vs 11 mg/dL, respectively; *P *< 0.0001), while exenatide caused a significantly greater reduction of the postprandial glucose (PPG) after breakfast (*P *< 0.0001) and dinner (*P *= 0.0005) but not lunch [13].

As add-on therapy, the FPG is reduced 11 to 29 mg/dL with the addition of a DPP-4 inhibitor to existing glucose-lowering therapy [48,50,51], with a greater reduction with sitagliptin compared to saxagliptin (16 mg/dL vs 11 mg/dL, respectively) [48]. The head-to-head comparison of liraglutide with sitagliptin showed that liraglutide reduced the FPG 31 to 37 mg/dL compared to 11 mg/dL for sitagliptin at 1 year (*P*≤0.0001) [46].

#### Postprandial glucose

Reduction in the A1C level observed with the GLP-1R agonists and DPP-4 inhibitors appears to result primarily from the generally greater reduction of the postprandial glucose (PPG) level compared to the FPG level (Table 1) [23,26,37,45,52]. A GLP-1R agonist appears preferred if the PPG level is significantly elevated, while a DPP-4 inhibitor may be preferred if there is modest elevation of both FPG and PPG [53].

Patients on stable doses of metformin and a sulfonylurea who were treated with exenatide 10 μg twice daily experienced significantly greater reduction in PPG excursions following breakfast and dinner than patients treated with insulin glargine once daily (P < 0.001) [54]. When once-daily liraglutide 1.2 mg or 1.8 mg was administered for 26 weeks as add-on therapy with metformin, the PPG level was reduced by 41 mg/dL and by 47 mg/dL, respectively, compared with a reduction of 45 mg/dL for glimepiride 4 mg once daily [55].

Treatment with sitagliptin 100 mg once daily for 18 weeks, when added to metformin, reduced the PPG by 68 mg/dL, compared with a reduction of 14 mg/dL for placebo [27]. Similarly, when saxagliptin is added to metformin therapy at doses of 2.5 mg once daily or 5 mg once daily, versus placebo, the PPG is reduced by 62 mg/dL, 58 mg/dL, and 18 mg/dL, respectively [9]. Linagliptin 5 mg once daily has been shown to reduce the PPG 49 mg/dL over 24 weeks compared to an increase of 18 mg/dL with placebo [51].

#### Effects on pancreatic β-cell function

Several clinical trials have examined the effects of the GLP-1R agonists and DPP-4 inhibitors on pancreatic β-cell function in persons with T2DM (Table 1). Since direct measurement of pancreatic β-cell function in humans is not possible, studies have measured a variety of surrogate markers. Improvement in some but not all markers has been observed in most studies involving the GLP-1R agonists [13,28,30,36,55,56] or DPP-4 inhibitors [9,50,57,58].

### Nonglycemic Effects

The effects of the GLP-1R agonists and DPP-4 inhibitors on nonglycemic parameters also serve to differentiate these agents from other glucose-lowering agents.

#### Body weight

Among the most important nonglycemic differences is the observation that patients treated with a GLP-1R agonist experienced a mean weight loss of 1 kg to 4 kg in clinical trials (Table 1) [29,37,39-41,49,52]. For example, after 3 years of exenatide treatment, 84% of patients lost weight, with 50% losing at least 5% of baseline body weight [59]. The amount of weight lost increased with higher body mass index [59]. The DPP-4 inhibitors are considered to be weight neutral; however, some patients experience a slight increase in weight, others a slight decrease [14,22,24,26,44,45,50,60]. The difference in weight effect between the GLP-1R agonists and DPP-4 inhibitors probably results from the ability of the GLP-1R agonists to reduce caloric intake by promoting satiety, and possibly by delaying gastric emptying [17,61]. The DPP-4 inhibitors do not appear to promote satiety or delay gastric emptying.

#### Lipids and blood pressure

While the GLP-1R agonists and DPP-4 inhibitors should not be used as primary therapy for cardiovascular disease, improvements in the lipid profile and reductions in blood pressure may offer additional benefits in an at-risk population with T2DM (Table 1). The mechanism for the lipid-lowering effect of the incretins is unknown, but may be related to their glucose-lowering effects as observed with other glucose-lowering agents or effects on free fatty acid metabolism. The greatest improvement in the lipid profile is observed in the triglyceride level, which is reduced by 12 to 40 mg/dL with the GLP-1R agonists [29,36,37,39,41,49,62], while the change with the DPP-4 inhibitors has ranged from an increase of 16 mg/dL to a decrease of 35 mg/dL [24,60]. Small changes in the LDL-cholesterol and HDL-cholesterol levels have been observed with both the GLP-1R agonists and the DPP-4 inhibitors.

The GLP-1R agonists also have been shown to lower systolic blood pressure 1 to 7 mm Hg; changes in diastolic blood pressure have been similar to placebo [13,29,36,37,39,41,49,62]. The mechanism for blood pressure reduction with the GLP-1R agonists is unclear. While an association with weight loss cannot be ruled out, one investigation involving liraglutide found that the reduction in systolic blood pressure occurred before substantial weight loss [29]. The effects on blood pressure with the DPP-4 inhibitors are limited [24,51,60].

### Safety and Tolerability

The good safety and tolerability of the GLP-1R agonists and DPP-4 inhibitors are well documented leading the AACE/ACE guidelines to conclude that the GLP-1R agonists are safer than sulfonylureas or glinides with respect to the risk of hypoglycemia [3]. While the guidelines also note the risk of gastrointestinal side effects (which are usually transitory) with the GLP-1R agonists and excellent tolerability of the DPP-4 inhibitors, some adverse events encountered in clinical practice bear discussion.

#### Hypoglycemia

A low incidence of hypoglycemia is observed with the GLP-1R agonists and DPP-4 inhibitors, likely because of their glucose-dependent actions on insulin and glucagon secretion (Table 1). For this reason, the ADA/EASD consensus statement recommends a GLP-1R agonist when hypoglycemia is a major concern, such as for those who have hazardous jobs and weight loss is beneficial [2]. Furthermore, the US Federal Aviation Administration includes the GLP-1R agonists and DPP-4 inhibitors in the list of allowable medications for aviators [63]. Severe hypoglycemia (generally defined as symptomatic--such as confusion, blurred vision, sweating--and requiring third-party assistance) has not been observed in trials of exenatide [36,52], liraglutide [37,38], sitagliptin [24,44], saxagliptin [26], or linagliptin [45] as monotherapy. Mild to moderate hypoglycemia (generally defined as asymptomatic or symptomatic with a blood glucose < 55 mg/dL but not requiring third-party assistance) occurs in 4% to 9% of patients receiving monotherapy treatment with exenatide [36,52], 0% to 12% with liraglutide [37,38], 0% to 4% with sitagliptin [22-24], 0-6% with saxagliptin [26], and 0% with linagliptin [45]. A slightly higher incidence of mild to moderate hypoglycemia is observed when incretin-based therapy is combined with 1 or more glucose-lowering agents [9,10,39,41,43,55,64]. However, when combined with a sulfonylurea, mild to moderate hypoglycemia has been reported by up to 36% of patients treated with the combination of a GLP-1R agonist or a DPP-4 inhibitor [14,65], which necessitates reducing the dose of the sulfonylurea, usually by half.

#### Nausea and Vomiting

Early experience with the GLP-1R agonists showed that transient nausea was common. This led to a dose titration strategy when initiating therapy, which reduced the incidence of transient nausea to 28% with exenatide and to 26% with liraglutide in a head-to-head comparison; vomiting occurred in 10% and 6%, respectively (Table 1) [13]. Exenatide should be initiated at a dose of 5 μg twice daily and taken within 60 minutes before the morning and evening meals; the dose can be increased to 10 μg twice daily after 1 month based on clinical response [31]. Liraglutide should be initiated independent of meals at a dose of 0.6 mg once daily for 1 week and then increased to 1.2 mg once daily. If the 1.2 mg dose does not result in acceptable glycemic control, the dose of liraglutide can be increased to 1.8 mg once daily to achieve glycemic control [32]. The incidence of nausea with the DPP-4 inhibitors is similar to placebo [22,23,26].

#### Acute pancreatitis

The possibility of acute pancreatitis, which was first raised by postmarketing reports with exenatide, led to changes in monitoring for its occurrence in subsequent clinical trials with incretin-based agents. These investigations show that acute pancreatitis has been observed rarely in patients treated with exenatide [31], liraglutide [32], and sitagliptin [33], and linagliptin [35]. For example, separate pooled analyses show that the number of cases of pancreatitis per 1000 patient-years was 2.2 for liraglutide (vs 0.6 for comparators) [32], 1.2 for sitagliptin (vs 1.0 for comparators) [33], and 1.7 for linagliptin (vs 0 for placebo) [35]. Similar pooled data for exenatide and saxagliptin are not available. However, establishing exenatide, liraglutide, sitagliptin, or linagliptin as the cause has not been possible because patients with T2DM regardless of treatment have a nearly 3-fold greater risk of pancreatitis compared with those without diabetes [66]. In addition, retrospective analysis of a health insurance database involving nearly 88,000 patients with 1-year follow-up demonstrated a similar risk of pancreatitis with exenatide, sitagliptin, metformin, and glyburide [67]. It is, nonetheless, important to educate patients about risk factors for pancreatitis, eg, gallstones, alcoholism, high triglycerides, and the immediate steps they should take if signs and symptoms suggestive of pancreatitis occur. At present, in patients with a history of pancreatitis, exenatide, liraglutide, and sitagliptin should not be prescribed [31-33], The FDA has required the manufacturers of exenatide, liraglutide, sitagliptin, saxagliptin, and linagliptin to conduct further epidemiologic studies of pancreatitis to clarify this issue [68-72].

#### Hypersensitivity reactions

Hypersensitivity reactions have been observed with each of the three DPP-4 inhibitors (Table 1). For sitagliptin, the most serious hypersensitivity reaction is Stevens-Johnson syndrome, which requires immediate treatment discontinuation if signs and symptoms of hypersensitivity occur [33]. Urticaria and facial edema occur in 1% to 2% of patients treated with saxagliptin [34], while urticaria, angioedema, localized skin exfoliation, or bronchial hyperreactivity can occur with linagliptin [35]. Further investigation of hypersensitivity reactions is ongoing with saxagliptin [72] and linagliptin [73], as mandated by the FDA.

#### Renal failure

Ischemic renal failure has been reported in four patients within two to nine months of starting exenatide [74]. All four patients presented with nausea, vomiting, or decreased fluid intake. In one patient, the renal failure was characterized by ischemic glomeruli with moderate to severe interstitial fibrosis, tubular atrophy, and early diabetic nephropathy. Recovery, which was incomplete in three of the patients upon cessation or dose reduction, was hypothesized to be due to volume contraction. Since exenatide, as well as sitagliptin and saxagliptin, are predominantly eliminated via the kidneys, the dose of these three agents must be reduced when the creatinine clearance is less than 50 mL/minute; exenatide is contraindicated if the creatinine clearance is less than 30 mL/minute [31,33,34]. Liraglutide and linagliptin do not require dosage adjustment in renal dysfunction [32,35].

#### Long-term Outcomes

The GLP-1R agonists and DPP-4 inhibitors have only become available in recent years, the first being exenatide in April 2005; thus, the long-term efficacy and safety of these 5 agents have not yet been established. Of the numerous ongoing investigations, several have been required by the FDA to clarify the long-term safety of these agents. One investigation relates to exenatide and a possible association between this agent and the development of thyroid cancer, based on postmarketing reports [69]. A second study is a preclinical investigation with liraglutide to determine the lifetime risk of developing thyroid C-cell tumors [70]. During review of the new drug application for liraglutide, the US FDA determined that there is a low risk of thyroid cancer in humans based on changes in rodents at levels of liraglutide many times those anticipated in humans [68]. Other investigation suggests that C-cell hyperplasia in rats and mice may be mediated by a GLP-1 receptor-mediated mechanism [75]. GLP-1 receptor expression in thyroid C-cells in humans and monkeys is low such that exposure to liraglutide at more than 60 times human exposure levels for 20 months did not result in C-cell hyperplasia in monkeys. Further, the level of calcitonin, a biomarker for medullary thyroid cancer, remained at the lower end of the normal range in humans exposed to liraglutide for 2 years. The FDA has required that an epidemiologic study of thyroid cancer be carried out with exenatide [69], as well as animal studies and monitoring a 15-year cancer registry with liraglutide [70]. In addition, a boxed warning concerning the current findings of medullary thyroid cancer in animals has been included in the prescribing information for liraglutide [32]. An association with sitagliptin [33] or saxagliptin [34] and thyroid cancer has not been identified.

Clinical trials investigating the cardiovascular effects of liraglutide [70], saxagliptin [72], and linagliptin [73] are also required (and are ongoing), since the clinical evaluations of these drugs were completed prior to December 2008 when the FDA adopted new standards regarding cardiovascular safety for all new antidiabetic drugs. These standards were in response to data suggesting a serious risk of cardiovascular events with some medications developed for the treatment of T2DM. Based on available data, the potential for adverse cardiovascular events with liraglutide, saxagliptin, and linagliptin cannot yet be definitively excluded.

These ongoing safety evaluations will better define the long-term safety of the GLP-1R agonists and DPP-4 inhibitors and identify safety issues, if any, earlier than previous and current postmarketing surveillance.

### Cost

In addition to efficacy and safety, cost is an important issue in selecting treatment and should be discussed with the patient. The purchase price for therapy with a GLP-1R agonist or DPP-4 inhibitor at a pharmacy is greater than for most other glucose-lowering agents. However, cost can vary substantially depending on formulary status and pharmacy. Also, patient insurance will affect the out-of-pocket cost to the patient since major costs are usually covered by insurance with a copay from the patient that is often dependent on formulary status. While the cost of a medication to the patient is a critical consideration in selecting therapy, the impact of a treatment on other direct and indirect costs also must be considered. Limited data have shown that the annual total cost of medical care ($19,293 vs $23,782, respectively; *P *< 0.0001) and total cost of diabetes-related medical care ($7,833 vs $8,536, respectively; *P *< .0001) with exenatide were significantly lower than with insulin glargine [76], including a lower cost related to the treatment of hypoglycemia [77]. Similarly, preliminary evidence indicates that liraglutide may reduce the total cost of diabetes-related care compared to glimepiride [78]. The analysis included costs due to ocular events and neuropathy leading to amputation. Although these data are preliminary, they are consistent with the clinical profile of the GLP-1R agonists and DPP-4 inhibitors, as discussed above.

## Conclusion

Following diagnosis of T2DM, glycemic control should be achieved within 3 to 6 months and maintained using a treat-to-target approach. Furthermore, treatment failure must not be accepted and must always be addressed quickly. Use of the GLP-1R agonists and DPP-4 inhibitors enables the primary care clinician to approach the management of patients with T2DM in a way that complements other therapies through the actions of these agents on the incretin pathway. This is especially important given that the incretin system is thought to be responsible for up to 70% of insulin secretion in response to oral glucose or a meal. Use of the GLP-1R agonists and DPP-4 inhibitors also enable the clinician to minimize the risk of some of the complications commonly encountered when treating patients with T2DM, such as hypoglycemia and weight gain. For these reasons, the GLP-1R agonists and DPP-4 inhibitors play an important role in the treatment of patients with T2DM, as reflected in recent consensus guidelines. But there are important differences among the incretin-based agents. The GLP-1R agonists require subcutaneous administration, produce pharmacological levels of GLP-1 activity, promote weight loss, have a more robust glucose-lowering effect, and have a higher incidence of adverse gastrointestinal effects. In contrast, DPP-4 inhibitors are taken orally, increase the half-life of endogenous GLP-1, are weight neutral, and are more commonly associated with nasopharyngitis. Although current evidence indicates these agents are safe and generally well-tolerated, the results of ongoing intensive safety evaluations will alert health care practitioners at the earliest possible time to important safety issues, should they occur.

## Competing interests

• Advisory board: Astra Zeneca, Bristol Myers Squibb, Genentech/Roche

•Speaker bureau: Abbott, Astra Zeneca, Bristol Myers Squibb, Eli Lilly, Forest, Kowa

## Authors' contributions

MC conceived of the article focus and scope, reviewed and revised the outline, reviewed, revised, and approved the final manuscript, and solely made the decision to submit the manuscript.

## References

[B1] Skyler JS Bergenstal R Bonow RO Buse J Deedwania P Gale EA Howard BV Kirkman MS Kosiborod M Reaven P Sherwin RS Intensive glycemic control and the prevention of cardiovascular events: implications of the ACCORD, ADVANCE, and VA diabetes trials: a position statement of the American Diabetes Association and a scientific statement of the American College of Cardiology Foundation and the American Heart Association Circulation 2009 119 351 357 10.1161/CIRCULATIONAHA.108.191305 19095622

[B2] Nathan DM Buse JB Davidson MB Ferrannini E Holman RR Sherwin R Zinman B Medical management of hyperglycemia in type 2 diabetes: a consensus algorithm for the initiation and adjustment of therapy: a consensus statement of the American Diabetes Association and the European Association for the Study of Diabetes Diabetes Care 2009 32 193 203 10.2337/dc08-9025 18945920 PMC2606813

[B3] Rodbard HW Jellinger PS Davidson JA Einhorn D Garber AJ Grunberger G Handelsman Y Horton ES Lebovitz H Levy P Moghissi ES Schwartz SS Statement by an American Association of Clinical Endocrinologists/American College of Endocrinology consensus panel on type 2 diabetes mellitus: an algorithm for glycemic control Endocr Pract 2009 15 540 559 19858063 10.4158/EP.15.6.540

[B4] Baggio LL Drucker DJ Biology of incretins: GLP-1 and GIP Gastroenterology 2007 132 2131 2157 10.1053/j.gastro.2007.03.054 17498508

[B5] Nauck MA Unraveling the science of incretin biology Am J Med 2009 122 S3 S10 19464426 10.1016/j.amjmed.2009.03.012

[B6] Naslund E Bogefors J Skogar S Gryback P Jacobsson H Holst JJ Hellstrom PM GLP-1 slows solid gastric emptying and inhibits insulin, glucagon, and PYY release in humans Am J Physiol 1999 277 R910 R916 10484511 10.1152/ajpregu.1999.277.3.R910

[B7] Fehmann HC Habener JF Insulinotropic hormone glucagon-like peptide-I(7-37) stimulation of proinsulin gene expression and proinsulin biosynthesis in insulinoma beta TC-1 cells Endocrinology 1992 130 159 166 10.1210/en.130.1.159 1309325

[B8] DeFronzo RA Okerson T Viswanathan P Guan X Holcombe JH MacConell L Effects of exenatide versus sitagliptin on postprandial glucose, insulin and glucagon secretion, gastric emptying, and caloric intake: a randomized, cross-over study Curr Med Res Opin 2008 24 2943 2952 10.1185/03007990802418851 18786299

[B9] DeFronzo RA Hissa MN Garber AJ Gross JL Duan RY Ravichandran S Chen RS for the Saxagliptin 014 Study Group The efficacy and safety of saxagliptin when added to metformin therapy in patients with inadequately controlled type 2 diabetes on metformin alone Diabetes Care 2009 32 1649 1655 10.2337/dc08-1984 19478198 PMC2732156

[B10] Charbonnel B Karasik A Liu J Wu M Meininger G Efficacy and safety of the dipeptidyl peptidase-4 inhibitor sitagliptin added to ongoing metformin therapy in patients with type 2 diabetes inadequately controlled with metformin alone Diabetes Care 2006 29 2638 2643 10.2337/dc06-0706 17130197

[B11] Kreymann B Williams G Ghatei MA Bloom SR Glucagon-like peptide-1 7-36: a physiological incretin in man Lancet 1987 2 1300 1304 2890903 10.1016/s0140-6736(87)91194-9

[B12] Nauck MA Heimesaat MM Behle K Holst JJ Nauck MS Ritzel R Hufner M Schmiegel WH Effects of glucagon-like peptide 1 on counterregulatory hormone responses, cognitive functions, and insulin secretion during hyperinsulinemic, stepped hypoglycemic clamp experiments in healthy volunteers J Clin Endocrinol Metab 2002 87 1239 1246 10.1210/jc.87.3.1239 11889194

[B13] Buse JB Rosenstock J Sesti G Schmidt WE Montanya E Brett JH Zychma M Blonde L for the LEAD-6 Study Group Liraglutide once a day versus exenatide twice a day for type 2 diabetes: a 26-week randomised, parallel-group, multinational, open-label trial (LEAD-6) Lancet 2009 374 39 47 10.1016/S0140-6736(09)60659-0 19515413

[B14] Chacra AR Tan GH Apanovitch A Ravichandran S List J Chen R Saxagliptin added to a submaximal dose of sulphonylurea improves glycaemic control compared with uptitration of sulphonylurea in patients with type 2 diabetes: a randomised controlled trial Int J Clin Pract 2009 63 1395 1406 10.1111/j.1742-1241.2009.02143.x 19614786 PMC2779994

[B15] Delgado-Aros S Kim DY Burton DD Thomforde GM Stephens D Brinkmann BH Vella A Camilleri M Effect of GLP-1 on gastric volume, emptying, maximum volume ingested, and postprandial symptoms in humans Am J Physiol Gastrointest Liver Physiol 2002 282 G424 G431 11841992 10.1152/ajpgi.2002.282.3.G424

[B16] Gutzwiller JP Goke B Drewe J Hildebrand P Ketterer S Handschin D Winterhalder R Conen D Beglinger C Glucagon-like peptide-1: a potent regulator of food intake in humans Gut 1999 44 81 86 10.1136/gut.44.1.81 9862830 PMC1760073

[B17] Gutzwiller JP Drewe J Goke B Schmidt H Rohrer B Lareida J Beglinger C Glucagon-like peptide-1 promotes satiety and reduces food intake in patients with diabetes mellitus type 2 Am J Physiol 1999 276 R1541 R1544 10233049 10.1152/ajpregu.1999.276.5.R1541

[B18] Holst JJ Deacon CF Glucagon-like peptide-1 mediates the therapeutic actions of DPP-IV inhibitors Diabetologia 2005 48 612 615 10.1007/s00125-005-1705-7 15759106

[B19] Herman GA Bergman A Stevens C Kotey P Yi B Zhao P Dietrich B Golor G Schrodter A Keymeulen B Lasseter KC Kipnes MS Snyder K Hilliard D Tanen M Cilissen C De Smet M de Lepeleire I Van Dyck K Wang AQ Zeng W Davies MJ Tanaka W Holst JJ Deacon CF Gottesdiener KM Wagner JA Effect of single oral doses of sitagliptin, a dipeptidyl peptidase-4 inhibitor, on incretin and plasma glucose levels after an oral glucose tolerance test in patients with type 2 diabetes J Clin Endocrinol Metab 2006 91 4612 4619 10.1210/jc.2006-1009 16912128

[B20] Zhou J Wang X Pineyro MA Egan JM Glucagon-like peptide 1 and exendin-4 convert pancreatic AR42J cells into glucagon- and insulin-producing cells Diabetes 1999 48 2358 2366 10.2337/diabetes.48.12.2358 10580424

[B21] Xu G Stoffers DA Habener JF Bonner-Weir S Exendin-4 stimulates both beta-cell replication and neogenesis, resulting in increased beta-cell mass and improved glucose tolerance in diabetic rats Diabetes 1999 48 2270 2276 10.2337/diabetes.48.12.2270 10580413

[B22] Aschner P Kipnes MS Lunceford JK Sanchez M Mickel C Williams-Herman DE Effect of the dipeptidyl peptidase-4 inhibitor sitagliptin as monotherapy on glycemic control in patients with type 2 diabetes Diabetes Care 2006 29 2632 2637 10.2337/dc06-0703 17130196

[B23] Raz I Hanefeld M Xu L Caria C Williams-Herman D Khatami H Efficacy and safety of the dipeptidyl peptidase-4 inhibitor sitagliptin as monotherapy in patients with type 2 diabetes mellitus Diabetologia 2006 49 2564 2571 10.1007/s00125-006-0416-z 17001471

[B24] Scott R Wu M Sanchez M Stein P Efficacy and tolerability of the dipeptidyl peptidase-4 inhibitor sitagliptin as monotherapy over 12 weeks in patients with type 2 diabetes Int J Clin Pract 2007 61 171 180 17156104 10.1111/j.1742-1241.2006.01246.x

[B25] Mari A Degn K Brock B Rungby J Ferrannini E Schmitz O Effects of the long-acting human glucagon-like peptide-1 analog liraglutide on beta-cell function in normal living conditions Diabetes Care 2007 30 2032 2033 10.2337/dc07-0310 17468345

[B26] Rosenstock J Sankoh S List JF Glucose-lowering activity of the dipeptidyl peptidase-4 inhibitor saxagliptin in drug-naive patients with type 2 diabetes Diabetes Obes Metab 2008 10 376 386 10.1111/j.1463-1326.2008.00876.x 18355324

[B27] Raz I Chen Y Wu M Hussain S Kaufman KD Amatruda JM Langdon RB Stein PP Alba M Efficacy and safety of sitagliptin added to ongoing metformin therapy in patients with type 2 diabetes Curr Med Res Opin 2008 24 537 550 10.1185/030079908X260925 18194595

[B28] Bunck MC Diamant M Corner A Eliasson B Malloy JL Shaginian RM Deng W Kendall DM Taskinen MR Smith U Yki-Jarvinen H Heine RJ One-year treatment with exenatide improves beta-cell function, compared with insulin glargine, in metformin-treated type 2 diabetic patients: a randomized, controlled trial Diabetes Care 2009 32 762 768 10.2337/dc08-1797 19196887 PMC2671094

[B29] Russell-Jones D Vaag A Schmitz O Sethi BK Lalic N Antic S Zdravkovic M Ravn GM Simo R on behalf of the Liraglutide Effect and Action in Diabetes 5 (LEAD-5) met-SU Study Group Liraglutide vs insulin glargine and placebo in combination with metformin and sulfonylurea therapy in type 2 diabetes mellitus (LEAD-5 met+SU): A randomised controlled trial Diabetologia 2009 52 2046 2055 10.1007/s00125-009-1472-y 19688338 PMC2744824

[B30] Pratley RE Nauck M Bailey T Montanya E Cuddihy R Filetti S Thomsen AB Sondergaard RE Davies M for the 1860-LIRA-DPP-4 Study Group Liraglutide versus sitagliptin for patients with type 2 diabetes who did not have adequate glycaemic control with metformin: a 26-week, randomised, parallel-group, open-label trial Lancet 2010 375 1447 1456 10.1016/S0140-6736(10)60307-8 20417856

[B31] Byetta [package insert] 2011 San Diego, CA, Amylin Pharmaceuticals, Inc.

[B32] Victoza [package insert] 2011 Princeton, NJ, Novo Nordisk Inc.

[B33] Januvia [package insert] 2011 Whitehouse Station, NJ, Merck & Co., Inc.

[B34] Onglyza [package insert] 2011 Princeton, NJ, Bristol-Myers Squibb Company

[B35] Tradjenta [package insert] 2011 Ridgefield, CT, Boehringer-Ingelheim Pharmaceuticals, Inc.

[B36] Moretto TJ Milton DR Ridge TD Macconell LA Okerson T Wolka AM Brodows RG Efficacy and tolerability of exenatide monotherapy over 24 weeks in antidiabetic drug-naive patients with type 2 diabetes: a randomized, double-blind, placebo-controlled, parallel-group study Clin Ther 2008 30 1448 1460 10.1016/j.clinthera.2008.08.006 18803987

[B37] Garber A Henry R Ratner R Garcia-Hernandez PA Rodriguez-Pattzi H Olvera-Alvarez I Hale PM Zdravkovic M Bode B for the LEAD-3 (Mono) Study Group Liraglutide versus glimepiride monotherapy for type 2 diabetes (LEAD-3 Mono): a randomised, 52-week, phase III, double-blind, parallel-treatment trial Lancet 2009 373 473 481 10.1016/S0140-6736(08)61246-5 18819705

[B38] Vilsboll T Zdravkovic M Le Thi T Krarup T Schmitz O Courreges JP Verhoeven R Buganova I Madsbad S Liraglutide, a long-acting human glucagon-like peptide-1 analog, given as monotherapy significantly improves glycemic control and lowers body weight without risk of hypoglycemia in patients with type 2 diabetes Diabetes Care 2007 30 1608 1610 10.2337/dc06-2593 17372153

[B39] Zinman B Hoogwerf BJ Garcia SD Milton DR Giaconia JM Kim DD Trautmann ME Brodows RG The effect of adding exenatide to a thiazolidinedione in suboptimally controlled type 2 diabetes: a randomized trial Ann Intern Med 2007 146 477 485 17404349 10.7326/0003-4819-146-7-200704030-00003

[B40] Kendall DM Riddle MC Rosenstock J Zhuang D Kim DD Fineman MS Baron AD Effects of exenatide (exendin-4) on glycemic control over 30 weeks in patients with type 2 diabetes treated with metformin and a sulfonylurea Diabetes Care 2005 28 1083 1091 10.2337/diacare.28.5.1083 15855571

[B41] Zinman B Gerich J Buse JB Lewin A Schwartz S Raskin P Hale PM Zdravkovic M Blonde L for the LEAD-4 Study Investigators Efficacy and safety of the human glucagon-like peptide-1 analog liraglutide in combination with metformin and thiazolidinedione in patients with type 2 diabetes (LEAD-4 Met+TZD) Diabetes Care 2009 32 1224 1230 10.2337/dc08-2124 19289857 PMC2699702

[B42] Rosenstock J Aguilar-Salinas C Klein E Nepal S List J Chen R Effect of saxagliptin monotherapy in treatment-naive patients with type 2 diabetes Curr Med Res Opin 2009 25 2401 2411 10.1185/03007990903178735 19650754

[B43] Rosenstock J Brazg R Andryuk PJ Lu K Stein P Efficacy and safety of the dipeptidyl peptidase-4 inhibitor sitagliptin added to ongoing pioglitazone therapy in patients with type 2 diabetes: a 24-week, multicenter, randomized, double-blind, placebo-controlled, parallel-group study Clin Ther 2006 28 1556 1568 10.1016/j.clinthera.2006.10.007 17157112

[B44] Hanefeld M Herman GA Wu M Mickel C Sanchez M Stein PP Once-daily sitagliptin, a dipeptidyl peptidase-4 inhibitor, for the treatment of patients with type 2 diabetes Curr Med Res Opin 2007 23 1329 1339 10.1185/030079907X188152 17559733

[B45] Del Prato S Barnett AH Huisman H Neubacher D Woerle HJ Dugi KA Effect of linagliptin monotherapy on glycaemic control and markers of beta-cell function in patients with inadequately controlled type 2 diabetes: a randomized controlled trial Diabetes Obes Metab 2011 13 258 267 10.1111/j.1463-1326.2010.01350.x 21205122

[B46] Pratley R Nauck M Bailey T Montanya E Cuddihy R Filetti S Garber A Thomsen AB Hartvig H Davies M for the 1860-LIRA-DPP-4 Study Group One year of liraglutide treatment offers sustained and more effective glycaemic control and weight reduction compared with sitagliptin, both in combination with metformin, in patients with type 2 diabetes: a randomised, parallel-group, open-label trial Int J Clin Pract 2011 65 397 407 10.1111/j.1742-1241.2011.02656.x 21355967 PMC3085127

[B47] Arnolds S Dellweg S Clair J Dain MP Nauck MA Rave K Kapitza C Further improvement in postprandial glucose control with addition of exenatide or sitagliptin to combination therapy with insulin glargine and metformin: a proof-of-concept study Diabetes Care 2010 33 1509 1515 10.2337/dc09-2191 20357372 PMC2890351

[B48] Scheen AJ Charpentier G Ostgren CJ Hellqvist A Gause-Nilsson I Efficacy and safety of saxagliptin in combination with metformin compared with sitagliptin in combination with metformin in adult patients with type 2 diabetes mellitus Diabetes Metab Res Rev 2010 26 540 549 10.1002/dmrr.1114 20824678

[B49] Blonde L Klein EJ Han J Zhang B Mac SM Poon TH Taylor KL Trautmann ME Kim DD Kendall DM Interim analysis of the effects of exenatide treatment on A1C, weight and cardiovascular risk factors over 82 weeks in 314 overweight patients with type 2 diabetes Diabetes Obes Metab 2006 8 436 447 10.1111/j.1463-1326.2006.00602.x 16776751

[B50] Hermansen K Kipnes M Luo E Fanurik D Khatami H Stein P Efficacy and safety of the dipeptidyl peptidase-4 inhibitor, sitagliptin, in patients with type 2 diabetes mellitus inadequately controlled on glimepiride alone or on glimepiride and metformin Diabetes Obes Metab 2007 9 733 745 10.1111/j.1463-1326.2007.00744.x 17593236

[B51] Taskinen MR Rosenstock J Tamminen I Kubiak R Patel S Dugi KA Woerle HJ Safety and efficacy of linagliptin as add-on therapy to metformin in patients with type 2 diabetes: a randomized, double-blind, placebo-controlled study Diabetes Obes Metab 2011 13 65 74 10.1111/j.1463-1326.2010.01326.x 21114605

[B52] Nelson P Poon T Guan X Schnabel C Wintle M Fineman M The incretin mimetic exenatide as a monotherapy in patients with type 2 diabetes Diabetes Technol Ther 2007 9 317 326 10.1089/dia.2006.0024 17705687

[B53] Rodbard HW Jellinger PS Davidson JA Einhorn D Garber AJ Grunberger G Handelsman Y Horton ES Lebovitz H Levy P Moghissi ES Schwartz SS AACE/ACE diabetes algorithm for glycemic control (update). American Association of Clinical Endocrinologists/American College of Endocrinology 2009 https://www.aace.com/sites/default/files/GlycemicControlAlgorithmPPT.pdf Accessed December 7, 2011 10.4158/EP.15.6.540 19858063

[B54] Heine RJ Van Gaal LF Johns D Mihm MJ Widel MH Brodows RG Exenatide versus insulin glargine in patients with suboptimally controlled type 2 diabetes: a randomized trial Ann Intern Med 2005 143 559 569 16230722 10.7326/0003-4819-143-8-200510180-00006

[B55] Nauck M Frid A Hermansen K Shah NS Tankova T Mitha IH Zdravkovic M During M Matthews DR for the LEAD-2 Study Group Efficacy and safety comparison of liraglutide, glimepiride, and placebo, all in combination with metformin, in type 2 diabetes: the LEAD (liraglutide effect and action in diabetes)-2 study Diabetes Care 2009 32 84 90 10.2337/dc08-1355 18931095 PMC2606836

[B56] Marre M Shaw J Brandle M Bebakar WM Kamaruddin NA Strand J Zdravkovic M Le Thi TD Colagiuri S on behalf of the LEAD-1 SU Study Group Liraglutide, a once-daily human GLP-1 analogue, added to a sulphonylurea over 26 weeks produces greater improvements in glycaemic and weight control compared with adding rosiglitazone or placebo in subjects with Type 2 diabetes (LEAD-1 SU) Diabet Med 2009 26 268 278 10.1111/j.1464-5491.2009.02666.x 19317822 PMC2871176

[B57] Brazg R Xu L Dalla MC Cobelli C Thomas K Stein PP Effect of adding sitagliptin, a dipeptidyl peptidase-4 inhibitor, to metformin on 24-h glycaemic control and beta-cell function in patients with type 2 diabetes Diabetes Obes Metab 2007 9 186 193 10.1111/j.1463-1326.2006.00691.x 17300594

[B58] Del Prato S Barnett AH Huisman H Neubacher D Woerle HJ Dugi KA Effect of linagliptin monotherapy on glycaemic control and markers of beta-cell function in patients with inadequately controlled type 2 diabetes: a randomized controlled trial Diabetes Obes Metab 2011 13 258 267 10.1111/j.1463-1326.2010.01350.x 21205122

[B59] Klonoff DC Buse JB Nielsen LL Guan X Bowlus CL Holcombe JH Wintle ME Maggs DG Exenatide effects on diabetes, obesity, cardiovascular risk factors and hepatic biomarkers in patients with type 2 diabetes treated for at least 3 years Curr Med Res Opin 2008 24 275 286 18053320 10.1185/030079908x253870

[B60] Hollander P Li J Allen E Chen R Saxagliptin added to a thiazolidinedione improves glycemic control in patients with type 2 diabetes and inadequate control on thiazolidinedione alone J Clin Endocrinol Metab 2009 94 4810 4819 10.1210/jc.2009-0550 19864452

[B61] Zander M Madsbad S Madsen JL Holst JJ Effect of 6-week course of glucagon-like peptide 1 on glycaemic control, insulin sensitivity, and beta-cell function in type 2 diabetes: a parallel-group study Lancet 2002 359 824 830 10.1016/S0140-6736(02)07952-7 11897280

[B62] Nauck MA Duran S Kim D Johns D Northrup J Festa A Brodows R Trautmann M A comparison of twice-daily exenatide and biphasic insulin aspart in patients with type 2 diabetes who were suboptimally controlled with sulfonylurea and metformin: a non-inferiority study Diabetologia 2007 50 259 267 10.1007/s00125-006-0510-2 17160407

[B63] Federal Aviation Administration Guide for aviation medical examiners. Pharmaceuticals (Therapeutic medications). Diabetes mellitus- type II, medication controlled (not insulin) 2011 http://www.faa.gov/about/office_org/headquarters_offices/avs/offices/aam/ame/guide/pharm/oral_diabetes/. Accessed December 7, 2011

[B64] DeFronzo RA Ratner RE Han J Kim DD Fineman MS Baron AD Effects of exenatide (exendin-4) on glycemic control and weight over 30 weeks in metformin-treated patients with type 2 diabetes Diabetes Care 2005 28 1092 1100 10.2337/diacare.28.5.1092 15855572

[B65] Buse JB Henry RR Han J Kim DD Fineman MS Baron AD Effects of exenatide (exendin-4) on glycemic control over 30 weeks in sulfonylurea-treated patients with type 2 diabetes Diabetes Care 2004 27 2628 2635 10.2337/diacare.27.11.2628 15504997

[B66] Noel RA Braun DK Patterson RE Bloomgren GL Increased risk of acute pancreatitis and biliary disease observed in patients with type 2 diabetes: a retrospective cohort study Diabetes Care 2009 32 834 838 10.2337/dc08-1755 19208917 PMC2671118

[B67] Dore DD Seeger JD Arnold CK Use of a claims-based active drug safety surveillance system to assess the risk of acute pancreatitis with exenatide or sitagliptin compared to metformin or glyburide Curr Med Res Opin 2009 25 1019 1027 10.1185/03007990902820519 19278373

[B68] Parks M Rosebraugh C Weighing risks and benefits of liraglutide--the FDA's review of a new antidiabetic therapy N Engl J Med 2010 362 774 777 10.1056/NEJMp1001578 20164475

[B69] Byetta. NDA approval. Supplemental approval US Food and Drug Administration 2009 http://www.accessdata.fda.gov/drugsatfda_docs/appletter/2009/021773s009s011s017s018s022s025021919ltr.pdf Accessed November 29, 2011

[B70] Victoza. NDA approval US Food and Drug Administration 2010 http://www.accessdata.fda.gov/drugsatfda_docs/nda/2010/022341s000approv.pdf Accessed November 29, 2011

[B71] Januvia. Supplement approval US Food and Drug Administration 2010 http://www.accessdata.fda.gov/drugsatfda_docs/appletter/2010/021995s010s011s012s014ltr.pdf Accessed November 29, 2011

[B72] Onglyza. NDA approval US Food and Drug Administration 2009 http://www.accessdata.fda.gov/drugsatfda_docs/appletter/2009/022350s000ltr.pdf Accessed November 29, 2011

[B73] Tradjenta. NDA Approval US Food and Drug Administration 2011 http://www.accessdata.fda.gov/drugsatfda_docs/appletter/2011/201280s000ltr.pdf Accessed November 29, 2011

[B74] Weise WJ Sivanandy MS Block CA Comi RJ Exenatide-associated ischemic renal failure Diabetes Care 2009 32 e22 e23 19171732 10.2337/dc08-1309 PMC6908415

[B75] Knudsen LB Madsen LW Andersen S Almholt K de Boer AS Drucker DJ Gotfredsen C Egerod FL Hegelund AC Jacobsen H Jacobsen SD Moses AC Molck AM Nielsen HS Nowak J Solberg H Thi TDL Zdravkovic M Glucagon-like Peptide-1 receptor agonists activate rodent thyroid C-cells causing calcitonin release and C-cell proliferation Endocrinology 2010 151 1473 1486 10.1210/en.2009-1272 20203154

[B76] Misurski D Lage MJ Fabunmi R Boye KS A comparison of costs among patients with type 2 diabetes mellitus who initiated therapy with exenatide or insulin glargine Appl Health Econ Health Policy 2009 7 245 254 19905038 10.1007/BF03256158

[B77] Fabunmi R Nielsen LL Quimbo R Schroeder B Misurski D Wintle M Wade R Patient characteristics, drug adherence patterns, and hypoglycemia costs for patients with type 2 diabetes mellitus newly initiated on exenatide or insulin glargine Curr Med Res Opin 2009 25 777 786 10.1185/03007990802715199 19203299

[B78] Sullivan SD Alfonso-Cristancho R Conner C Hammer M Blonde L A simulation of the comparative long-term effectiveness of liraglutide and glimepiride monotherapies in patients with type 2 diabetes mellitus Pharmacotherapy 2009 29 1280 1288 10.1592/phco.29.11.1280 19873688

[B79] Buse JB Garber A Rosenstock J Schmidt WE Brett JH Videbaek N Holst J Nauck N Liraglutide treatment is associated with a low frequency and magnitude of antibody formation with no apparent impact on glycemic response or increased frequency of adverse events: results from the Liraglutide Effect and Action in Diabetes (LEAD) trials J Clin Endocrinol Metab 2011 96 1695 1702 10.1210/jc.2010-2822 21450987

[B80] Amori RE Lau J Pittas AG Efficacy and safety of incretin therapy in type 2 diabetes: systematic review and meta-analysis JAMA 2007 298 194 206 10.1001/jama.298.2.194 17622601

